# Spinal cord perfusion pressure protocol for acute spinal cord injury: Pragmatic implementation and early results at two sites

**DOI:** 10.1371/journal.pone.0341863

**Published:** 2026-01-30

**Authors:** John Paul G. Kolcun, Ricky M. Ditzel Jr, Bradley L. Kolb, Ricardo B. V. Fontes, P. B. Raksin

**Affiliations:** 1 Department of Neurological Surgery, Rush University Medical Center, Chicago Illinois, United States of America; 2 Division of Neurological Surgery, John H. Stroger Hospital of Cook County, Chicago Illinois, United States of America; Nationwide Children's Hospital, UNITED STATES OF AMERICA

## Abstract

**Study design:**

Retrospective chart review.

**Objective:**

Describe safety/feasibility of implementing a novel clinical protocol for acute spinal cord injury (SCI) management.

**Summary of background data:**

Spinal cord perfusion pressure (SCPP) has emerged as a promising target for the medical management of SCI patients. We report our early experience implementing a pragmatic SCPP-driven clinical protocol to supplant conventional mean arterial pressure (MAP) monitoring in the setting of acute SCI.

**Methods:**

We retrospectively reviewed charts of all SCI patients managed by our SCPP protocol since its adoption at two clinical sites as of 2/1/2023. The SCPP protocol was applied for all adult SCI patients of any injury grade, at any injury level with cord tissue involvement. Intrathecal pressure (ITP) was transduced by lumbar drain (LD). MAP was determined from invasive blood pressure recordings. SCPP was calculated as the difference between MAP and ITP, with an SCPP goal of >65mmHg.

**Results:**

Eighteen patients have been treated since our SCPP protocol was adopted. Patients were predominately male (77.8%); the average age was 52.0 ± 16.2 years. Most injuries involved the cervical segment (72.2%), all of which were manifest clinically as central cord syndrome. The most common presenting injury severity was ASIA Impairment Scale D (44.4%).

All patients underwent surgical intervention. There were no complications related to surgery, LD placement, or LD maintenance/ITP transduction during hospitalization. The SCPP protocol was continued for an average 5.2 ± 1.8 days. Eight patients required vasopressor support (44.4%) during that period, for an average 3.1 ± 2.1 days. Five patients underwent therapeutic CSF drainage to augment SCPP (27.8%). All patients maintained an average SCPP above goal for the duration of monitoring.

**Conclusions:**

This study further establishes the safety and feasibility of monitoring SCPP via LD measurement of ITP in acute SCI patients treated by clinical protocol at two clinical sites. There were no complications related to LD placement/maintenance or SCPP monitoring.

## Introduction

Traumatic spinal cord injury (SCI) remains a significant global burden, impacting patients, families, and caregivers [1–3]. While these are historically injuries of the young, recent demographic trends (particularly in developed nations) suggest an increasing incidence of central cord syndrome (CCS). CCS disproportionately affects older patients with underlying degenerative cervical stenosis and possibly a baseline degenerative myelopathy. In fact, CCS may now be the most common form of SCI [4,5]. As a result, we now face an expanding and heterogenous population of SCI patients with variable injury mechanisms, medical co-morbidities, and physiologic reserves.

The optimal treatment of acute, traumatic SCI was the focus of extensive research in the late 20^th^ Century. After decades of investigation and controversy, two interventions have remained a consistent part of the discussion: i) consideration of early surgical intervention [6]; and ii) maintenance of an elevated mean arterial pressure (MAP) in the early post-injury period [7]. In addition to relieving direct mechanical distortion by early decompression (as in the case of a fracture-dislocation, for example), these interventions share a common goal: to ensure adequate perfusion to injured spinal cord tissue and surrounding viable tissue at risk of secondary injury.

More recently, spinal cord perfusion pressure (SCPP) has been studied as an alternative to MAP goals for the post-injury medical management of SCI patients. SCPP is defined as the difference between MAP and intrathecal pressure (ITP), as transduced via lumbar drain (LD) or intrathecal pressure probe (SCPP = MAP – ITP) [8,9]. Yue et al. recently published their experience after fully transitioning their center from management by MAP goals to a SCPP-based protocol [10]. This is the only published report to-date of a center transitioning to SCPP management as standard clinical practice, outside of a clinical trial.

In early 2023, our department adopted a pragmatic SCPP clinical protocol for acute SCI management in place of conventional MAP goals. We report our early experience after pragmatically implementing this clinical protocol outside of a clinical trial, with a focus on SCPP-related interventions, monitoring, complications, and clinical outcomes during the index hospitalization.

## Materials & methods

We retrospectively reviewed charts of all patients who presented with SCI and were treated according to our SCPP protocol from 2/1/2023 to the present at two associated sites: a public, level-one trauma center and a private, academic level-two center. The sites are adjacent to one another in a major urban area. The SCPP protocol was implemented at both centers on 2/1/2023, with the agreed participation of all associated services providing intensive care for these patients. From that date forward, any patient with acute, closed SCI who would otherwise be managed by goal MAP parameters was instead managed by SCPP goals, as transduced by lumbar drain.

Specifically, we included all adult patients (defined as ≥ 16 years of age for trauma patients at our center) who presented with acute, closed SCI within 24 hours from injury, with any American Spinal Injury Association Impairment Scale (AIS) grade of injury (AIS A/B/C/D, including central cord syndrome), at any spinal segment involving cord tissue (C0-L2, typically). We excluded patients with penetrating SCI and/or a medical or surgical contraindication to LD placement. This cohort would still be managed by conventional MAP goals at the discretion of the attending surgeon.

Our pragmatic protocol calls for LD placement at the time of surgery or preoperatively at bedside if surgical treatment is anticipated to occur outside of the initial 24-hour admission window for any reason. Preoperative LD were placed in the lateral decubitus position, in using standard technique for bedside placement, with additional precautions for spinal/cervical stability as needed. Intraoperative LD were placed either in lateral decubitus (for patients undergoing anterior surgery) or in the prone position on the operative table at the end of surgery. Opening pressures were measured at the time of LD placement. From the time of LD placement, we set an SCPP goal range of 65–100 mmHg. To determine SCPP, we lay the patient flat each hour, re-zero the LD transducer at the phlebostatic axis, and measure the ITP. This ITP reading sets the “hourly MAP goal” necessary to maintain SCPP within the target range. Thus, SCPP is manipulated primarily by MAP control. Therapeutic cerebrospinal fluid diversion (CSFD) for ITP manipulation is not an explicit part of our current institutional protocol but is available as an option after surgical decompression at the discretion of the attending neurosurgeon, generally to bring the ITP < 15mmHg in the setting of reduced SCPP with a high/normal MAP/SBP and an elevated ITP (e.g., SCPP 50, MAP 85, ITP 35). Similarly, the duration of adherence to the SCPP protocol was left to the discretion of the attending neurosurgeon but typically was followed for 5 days (reflecting each physician’s common practice for convention MAP goals). Corticosteroid administration is not practiced at either institution for acute SCI, and none were administered to patients included in this study. Likewise, prophylactic antibiotics are not used with LD in place at either institution.

We collected data regarding patient demographics, injury mechanism, clinical presentation, perioperative medical interventions, LD placement, surgical interventions, SCPP/MAP management, and overall hospital course/discharge.

Data were collected on multiple dates spanning 01/11/2024–01/03/2025. Senior authors had access to identifiable data for record-keeping during the study. Continuous variables are reported as *mean ± standard deviation*; categorical variables are reported as *N (%)*.

## Results

We identified 18 patients treated under the SCPP protocol. The average age of included patients was 52.0 ± 16.2; four patients were female (22.2%). The most common mechanism of injury was a fall from height (13, 72.2%). Most cases were seen at our public, level-one trauma center (14, 77.8%). Medical co-morbidities included baseline myelopathy (7, 38.9%), vasculopathy (5, 27.8%), and diabetes (5, 27.8%). Five patients had concomitant non-neurologic injuries (27.8%) (Table 1).

**Table 1:** Sample characteristics.

No. patients	18		
Female	4 (22.2)		
Age (years)	52.0 ± 16.2		
Myelopathy	7 (38.9)		
		
Vasculopathy*	5 (27.8)		
DM	5 (27.8)		
Associated injury	5 (27.8)		
*Spinal region of injury*			
Cervical	13 (72.2)		
Thoracic	4 (22.2)		
Lumbar	1 (5.6)		
*Associated neurologic findings*			
CCS	13 (72.2)		
Sphincter tone	16 (88.9)		
Dysesthesia	10 (55.6)		
*AIS grade*	*At presentation*	*At 72 hours*	*At discharge*
A	3 (16.7)	2 (11.1)	2 (11.1)
B	1 (5.6)	2 (11.1)	2 (11.1)
C	6 (33.3)	3 (16.7)	1 (5.6)
D	8 (44.4)	11 (61.1)	12 (66.7)
E	0	0	1 (5.6)

DM: diabetes mellitus; CCS: central cord syndrome; AIS: American Spinal Injury Association Impairment Scale; *Vasculopathy here represents a composite of peripheral vascular disease and coronary artery disease.

*Continuous variables are shown as mean ±standard deviation; categorical variables as N (%)*.

Most patients presented with cervical injuries; all cervical cases presented with a spinal cord injury without radiological instability, usually referred to as central cord syndrome (13, 72.2%). The remaining injuries involved the thoracic spine (4, 22.2%), and one occurred at the first lumbar segment. The most common AIS grade at presentation, at 72 hours after presentation, and at discharge was D. Almost all patients had preserved sphincter tone (16, 88.9%), and 10 experienced burning dysesthesia (55.6%) (Table 1).

All patients were treated surgically, as is the standard practice in our group: 5 anteriorly (27.8%) and 13 posteriorly (72.2%). On average, surgery occurred 30.9 ± 30.8 hours after presentation to one of our clinical sites. There were no surgical complications. LD placement occurred preoperatively in 7 cases (38.9%), and intraoperatively in 10 cases (55.6%). In one patient, LD placement during surgery was unsuccessful, so it was placed postoperatively in the interventional radiology suite. Opening pressure was recorded in 9 cases with an average value of 18.2 ± 5.5 cmH_2_O. There were no procedural complications related to LD placement (Table 2).

**Table 2:** Treatment course.

Time to surgery (hours)	30.9 ± 30.8
*Surgical approach*	
Anterior	5 (27.8)
Posterior	13 (72.2)
*LD timing*	
Pre-op	7 (38.9)
Intra-op	10 (55.6)
Post-op	1 (5.6)
Opening pressure (cmH2O)	18.2 ± 5.5
LD complications	0
No. SCPP days	5.2 ± 1.8
Vasopressor use	8 (44.4)
No. vasopressor days	3.1 ± 2.1
CSFD	5 (27.8)

*LD: lumbar drain; SCPP: spinal cord perfusion pressure: CSFD: cerebrospinal fluid diversion; “Time to surgery” represents time from presentation at our hospital to surgery start time*.

*Opening pressure was taken in 9 of 18 total cases*.

*Continuous variables are shown as mean ±standard deviation; categorical variables as N (%)*.

The duration of SCPP monitoring was an average of 5.2 ± 1.8 days (range 2–9 days). Eight patients required vasopressor support (44.4%) during that period. In these cases, the average duration of vasopressor requirement was 3.1 ± 2.1 days. Therapeutic CSFD was utilized in 5 cases (27.8%). There were no device-related complications related to the LD, SCPP transduction, or therapeutic CSFD. In one patient, the period of SCPP monitoring was truncated due to concern for an occult CSF leak related to the trauma. In this case, the LD was utilized to control the leak with conventional hourly CSFD goals, and we reverted to conventional MAP goals for SCI management (Table 2).

Eight patients had a medical complication during their hospital course (44.4%), including urinary tract infection (3, 16.7%), pneumonia (2, 11.1%), and cardiac/myocardial infarction (2, 11.1%). Average hospital length of stay was 18.7 ± 12.9 days. Most patients were discharged to an in-patient facility (13, 72.2%), while 5 were discharged directly home (27.8%). No patients reported headache or other symptoms related to CSFD (Table 3).

**Table 3:** Hospital course.

Hospital complication (no. patients)	8 (44.4)
UTI	3 (16.7)
Cardiac/MI	2 (11.1)
PNA	2 (11.1)
LOS (days)	18.7 ± 12.9
Home discharge	5 (27.8)
Facility discharge	13 (72.2)

*UTI: urinary tract infection; MI: myocardial infarction; PNA: pneumonia; LOS: length of stay*.

*Continuous variables are shown as mean ±standard deviation; categorical variables as N (%)*.

**Fig 1:**
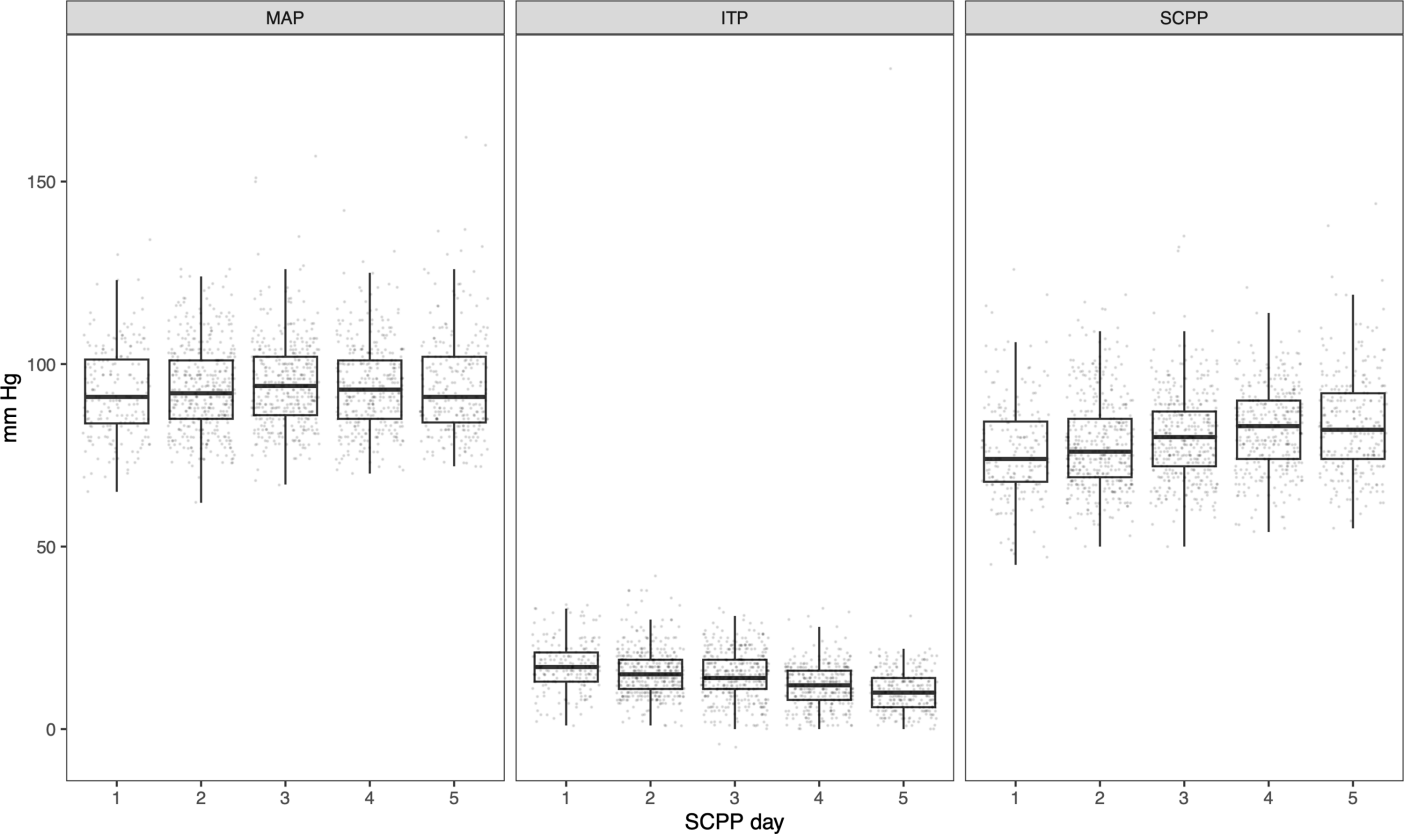
Standard box-and-whisker plots depicting summary statistics for daily recorded values of MAP, ITP, and SCPP. Individual measurements are shown as dots. Thick lines depict median values, boxes depict interquartile ranges (IQR), and whiskers depict lowest and highest data points within 1.5 times the IQR. (Data are shown for SCPP days 1-5, as only 3 patients underwent SCPP monitoring for >5 days; full data beyond SCPP day 5 are available upon request). *MAP: mean arterial pressure; ITP: intrathecal pressure; SCPP: spinal cord perfusion pressure.*

**Fig 2:**
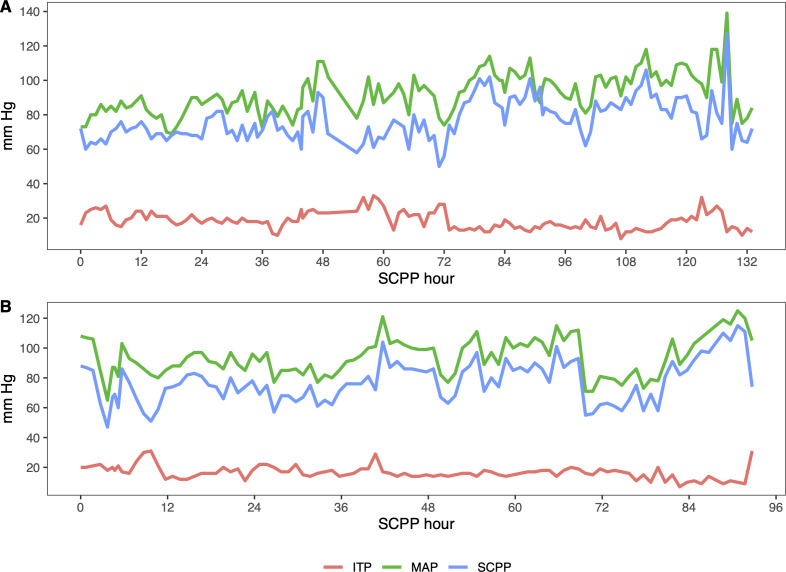
Hourly pressures during SCPP protocol for A: Patient 2, who sustained a T3 AIS A SCI after a fall, and B: Patient 8, who sustained a C6 AIS D SCI with central cord syndrome after a fall. MAP: mean arterial pressure; ITP: intrathecal pressure; SCPP: spinal cord perfusion pressure.

**Fig 3:**
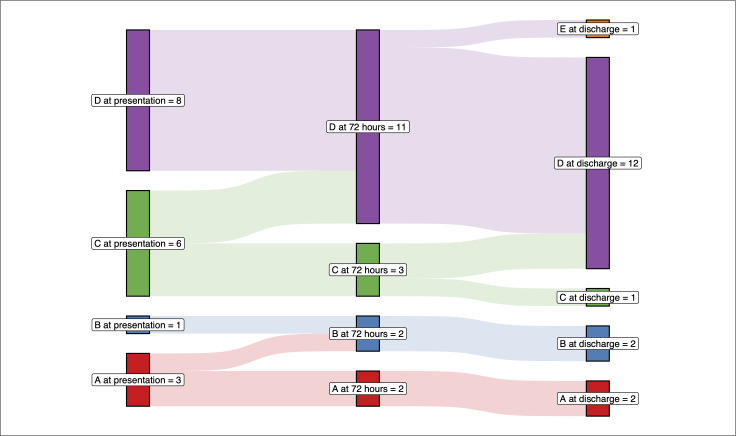
Sankey diagram AIS grades at presentation, 72 hours post-injury, and at discharge for the cohorts, including AIS conversions between time-points. The width of each band is proportional to the number of patients represented at each point. *AIS: American Spinal Injury Association Impairment Scale.*

**Fig 4:**
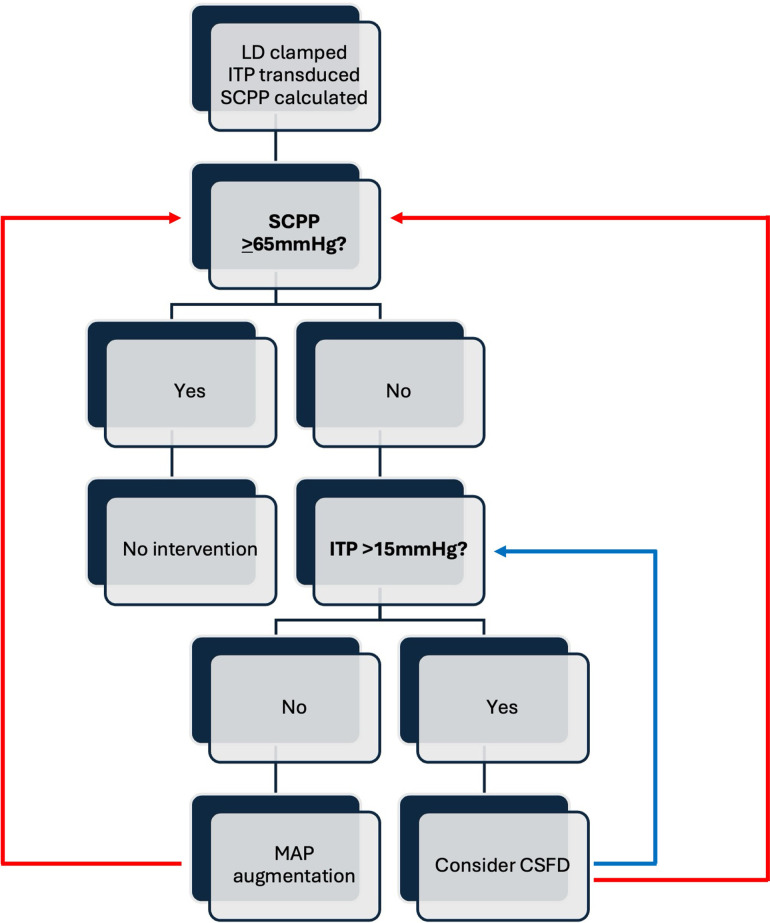
A decision-tree displaying our general approach to the pragmatic SCPP protocol, indicating when to consider MAP augmentation vs. CSFD to bring SCPP to the goal range. LD: lumbar drain; ITP: intrathecal pressure; SCPP: spinal cord perfusion pressure; CSFD: cerebrospinal fluid diversion.

All patients maintained average SCPP within target range for the duration of SCPP monitoring. Details of individual patients’ presentation and average SCPP across the monitoring period are shown in Table 4. Average daily MAP, ITP, and SCPP for the entire cohort are shown in Table 5. Daily transduced MAP and ITP, and calculated SCPP, are shown on average for the population in Fig 1. Fig 2 shows hourly MAP, ITP, and SCPP across the monitoring period for two representative cases. AIS grade at presentation, at 72 hours, and at time of discharge for the cohort (including conversions between time points) are shown in Fig 3. Fig 4 shows a general decision-tree diagram for consideration of CSFD vs. MAP augmentation alone for SCPP support.

**Table 4:** Individual case details.

Patient	Age (years)	MOI	AIS grade at presentation	SCI level	Time to surgery (hours)	LD timing	CSFD	No. SCPP days	No. Pressor days	Overall SCPP
1	67	Fall	D	C8	18	Intra-op	–	4	1	86.9 ± 20.9
2	39	Fall	A	T3	6	Post-op	Yes	6	5	76.9 ± 11.8
3	20	Motorcycle accident	C	T4	12	Intra-op	–	9	5	71.3 ± 8.1
4	69	Fall	D	C5	33	Pre-op	–	4	–	82.1 ± 10.7
5	38	Fall	D	C7	54	Pre-op	–	2	–	91.8 ± 7.5
6	44	Fall	D	C7	27	Pre-op	–	5	–	94.5 ± 12.1
7	52	Fall/assault	D	C7	23	Pre-op	–	3	–	74.8 ± 8.2
8	74	Fall	D	C6	59	Intra-op	–	4	–	77.1 ± 14.0
9	40	Fall	C	C3	35	Intra-op	–	7	3	75.6 ± 9.1
10	75	Fall	C	C5	16	Intra-op	–	5	3	75.6 ± 11.5
11	58	Fall	C	T11	3	Intra-op	–	4	0	80.4 ± 9.9
12	35	Jump from overpass	A	L1	64	Pre-op	–	5	5	76.6 ± 9.1
13	63	Fall	D	C5	99	Pre-op	–	4	–	89.2 ± 9.8
14	70	Fall	C	C6	3	Intra-op	Yes	5	4	72.0 ± 7.3
15	46	MVC	D	C5	93	Pre-op	–	5	–	81.4 ± 12.1
16	31	Fall	A	T8	1	Intra-op	Yes	8	5	78.8 ± 9.3
17	55	Bicycle accident	C	C6	4	Intra-op	Yes	7	0	83.8 ± 8.2
18	60	Fall	B	C4	6	Intra-op	Yes	7	0	79.2 ± 11.3

*MOI: mechanism of injury; AIS: American Spinal Injury Association Impairment Scale; SCI: spinal cord injury; LD: lumbar drain; CSFD: cerebrospinal fluid diversion; SCPP: spinal cord perfusion pressure; “Time to surgery” represents time from presentation at our hospital to surgery start time*.

*Continuous variables are shown as mean ±standard deviation*.

**Table 5:** Cohort average pressures during SCPP protocol. (Average values are shown for SCPP days 1-5, as only 6 of 18 patients underwent SCPP monitoring for >5 days; full data beyond SCPP day 5 are available upon request).

SCPP day	MAP	ITP	SCPP
1	92.0 ± 14.0	17.3 ± 6.9	76.0 ± 14.7
2	93.4 ± 11.8	15.5 ± 6.5	78.1 ± 12.5
3	94.8 ± 12.9	14.6 ± 6.5	80.0 ± 11.7
4	93.9 ± 11.8	11.9 ± 5.8	82.4 ± 11.0
5	94.0 ± 14.1	11.1 ± 11.5	83.7 ± 13.8

*SCPP: spinal cord perfusion pressure; MAP: mean arterial pressure; ITP: intrathecal pressure*.

*Continuous variables are shown as mean ±standard deviation*.

## Discussion

This is the second published report documenting an institution’s transition from targeting MAP goals to SCPP to guide the medical management of acute SCI as standard clinical practice, outside a clinical trial or other research setting. We describe our early experience with the implementation of this protocol at two sites with very different patient populations (one public, one private) and show that SCPP management via LD transduction was safe and feasible.

Prior studies have raised concerns about the suitability of MAP goals as the optimal management strategy after acute SCI, citing unobservable hemodynamic changes in the intrathecal/intraparenchymal environment at the site of injury and in surrounding viable tissue, variable responses to different vasopressor agents within the intrathecal space with inconsistent effects on actual SCPP, and the significant side effects associated with vasopressor administration in older, frailer patients (who comprise an increasing proportion of SCI patients) [11–13]. Indeed, the recommended threshold for MAP goals was originally established by intuition more than evidence [14]. SCPP offers the possibility of a dynamic physiologic parameter measured in real time and tailored to the need of individual patients.

Emerging evidence favors SCPP over MAP in predicting neurologic recovery after SCI. In a prospective observational cohort study, Squair et al. showed that acute SCI patients who experienced low SCPP (<50mmHg, as transduced by LD) were significantly less likely to recover one AIS grade at 6 months after injury; in this study, MAP or ITP alone were not predictive of neurologic recovery [8]. Saadoun et al. similarly showed that SCPP was correlated with neurologic improvement at 9–12 months; however, this group transduced ITP via a pressure probe inserted intrathecally at the time of surgical decompression [9]. Both methods are effective for ITP transduction, each with relative merits.

Our center chose LD placement for ITP measurement for the following reasons: i) LD placement can be performed at the bedside preoperatively, intraoperatively, or postoperatively at any time; ii) LD placement enables the possibility of therapeutic CSFD; and iii) intrathecal pressure probes have been associated with minor procedural/device complications (though few), whereas LD placement in the setting of SCI has not been associated – to date – with any procedural or device-related complication [15,16]. Prior studies have shown discordance between ITP transduced by LD in the lumbar cistern versus ITP measured directly at the site of injury, as well as discordance between transduced ITP and intraparenchymal pressure at the site of injury, with an average difference of ~10–15 mmHg between the injury site and the lumbar cistern ITP [17,18]. This effect would of course be pronounced in cases of severe, occlusive cord swelling at the injury site, creating a discontinuity in CSF flow and thus the pressure wave transmitted to the LD. To monitor for this effect, we would routinely evaluate LD function, including CSF flow and ITP waveform morphology and respiratory variation. With these considerations, and given that Squair et al. nevertheless demonstrated that SCPP – as determined by LD – was more predictive of neurologic recovery than MAP or ITP alone, we felt confident that using pressures measured by LD and setting our SCPP goal above the critical threshold of 50 mmHg derived in that study would provide a reasonable surrogate, whether or not the pressures transduced at the lumbar cistern accurately reflect those at the injury site. We thus chose a lower SCPP threshold of 65 mmHg, as did Yue et al [10]. We set a *pro forma* upper SCPP threshold of 100 mmHg to address variability concerns from intensive care and nursing staff. We did not experience issues with nursing burden or compliance at either institution; at both hospitals, nursing staff frequently care for patients with external ventricular drains, which require hourly checks, re-leveling, and pressure recording, and so were not unduly burdened by similar hourly management for SCPP patients. We also experienced no issues with LD placement, including during positioning, likely due to the fact that the majority of LD were placed intraoperatively, with the patient already safely positioned for surgery. The timing and method of LD placement should always be tailored to the individual patient, particularly in cases of extreme rotational instability.

Therapeutic CSFD was used in 5 cases in our series. All cases experienced low SCPP refractory to further MAP elevation in the setting of elevated ITP during the early post-operative period. In a prospective, randomized trial, Kwon et al. randomized acute SCI patients to LD placement with or without CSFD [19]. They found minimal effect of CSFD on ITP/SCPP, although the sample size and the volume of CSFD were both small. They also observed no adverse events related to pre-decompression LD placement or post-decompression CSFD in these patients. More recently, Theodore et al. completed a prospective randomized trial in a small cohort of cervical SCI patients with LD placement and elevated MAP goals, randomized to CSFD with goal ITP < 10 mmHg or no CSFD [20]. With this active therapeutic drainage regimen, they observed lower ITP, higher SCPP, and greater neurologic improvement in the CSFD cohort. Furthermore, Martirosyan et al. demonstrated that actual spinal cord blood flow (directly observed in an animal model) was significantly increased in the setting of MAP augmentation in combination with CSFD, to a greater extent than either MAP augmentation or CSFD alone [21]. Finally, there is an existing body of literature from vascular surgery that clearly demonstrates the power of CSFD to improve spinal cord perfusion by reduced ITP in the setting of hypovolemia [22]. While imperfectly analogous to acute SCI, this suggests that CSFD is at least an effective tool for physiologic manipulation of this compartment: it is not yet clear whether this will translate into demonstrable neurologic recovery for SCI patients. CSFD for ITP/SCPP manipulation in SCI is a nascent field of research, but these studies provide foundational evidence of safety and feasibility for future investigation.

Finally, the authors note that there were no patients who denied consent for LD placement for SCPP monitoring. As this was a clinical protocol implemented outside of a research or trial context, we presented LD placement and SCPP monitoring as a novel, but safe and reasonable part of our treatment plan, including the basic physiology and rationale for doing so. As we implement and advocate for other centers to adopt a new treatment paradigm in this population, we feel it is important to emphasize that in our experience thus far, patients have been agreeable to this novel intervention.

### Limitations

This is a retrospective study at two affiliated centers in the same metropolitan area; though located in close geographic proximity, these centers serve very different patient populations. The influence of demographic differences with respect to race, socioeconomic status, and comorbidities on outcomes may be significant, but is outside the scope of the current project.

Our series contains a high percentage of CCS patients, whose pathophysiology, hemodynamics, and overall natural history may differ significantly from other traumatic SCI patients. However, as CCS has increased in incidence and proportion of overall SCI in recent years, this reflects an observed trend in the population of SCI patients. All patients in this cohort were treated surgically, including those with CCS. This is the standard practice at our centers, and the surgical decision-making for these patients was in no way altered by our transition to SCPP management in the perioperative period. Likewise, there was no variation in SCPP protocol for patients with CCS. We hope that transitioning our practice to SCPP targeting in place of MAP goals will reduce the administration of potentially unnecessary vasopressors in these patients, who are often elderly with poor cardiac function, but present study is not equipped to answer this question.

It is also important to note that this “first-pass” protocol intentionally included room for variation in the execution of certain elements including the timing of LD placement, the duration of SCPP monitoring, and the decision to incorporate therapeutic CSFD. This was a pragmatic decision to gain the trust and encourage the comfort of participating physicians, and ease the transition to SCPP-based management. It is our hope that as we gain further experience with this protocol, the process can be further refined, and there will be less variability among physicians. In this regard, however, our SCPP management reflects the real-world variability in MAP management for SCI patients, which may be different across institutions or managing physicians. As our transition to SCPP management was a shift in institutional clinical practice, outside of a formal clinical trial or other research setting, variability across physicians was expected, and thus pragmatically incorporated into our overall clinical paradigm for SCPP management.

The retrospective nature of the study further precludes higher-granularity review of certain data, such as vasopressor administration and medium- to long-term clinical follow-up. Additionally, because our medical records do not consistently capture the actual time of injury, we can only report time from hospital presentation to surgery, and acknowledge that there may be significant variability in the interval from injury to presentation (particularly if a transfer was involved). The significance of this variability with respect to outcome is uncertain. However, it remains that the focus of the present study is the feasibility and safety of SCPP protocol implementation rather than outcomes; future studies can address these questions.

Finally, we again acknowledge the concern that pressure transduction at the lumbar cistern may not accurately reflect actual ITP at the site of injury; it is unclear whether this measurement discordance is clinically significant. An analogy might be the use of an intraparenchymal pressure probe versus an external ventricular drain for intracranial pressure monitoring, where the former is a necessarily regional and the latter more global measure, though both are accepted for monitoring. It follows that this difference may be more relevant prior to surgical decompression, when mechanical canal compromise or cord swelling may impede the communication of pressure waves between the segments of the spinal canal rostral/caudal to the injury site. Our use of LD transduction and our lower threshold for SCPP goals reflect those previously reported in non-research clinical practice.

## Conclusions

We report the successful transition from management by MAP goals to spinal cord perfusion pressure parameters for the management of acute spinal cord injury across two centers: a public level-one hospital and a private, academic level-two hospital. The SCPP protocol, relying upon ITP measurement by LP and coupled with invasive blood pressure measurement, was implemented without complication. Future studies may address optimal SCPP target range and duration of monitoring, as well as the influence of therapeutic cerebrospinal fluid drainage on functional outcomes.
